# Intradermal immunisation using the TLR3-ligand Poly (I:C) as adjuvant induces mucosal antibody responses and protects against genital HSV-2 infection

**DOI:** 10.1038/npjvaccines.2016.10

**Published:** 2016-08-25

**Authors:** Emilie Bardel, Remi Doucet-Ladeveze, Cyrille Mathieu, Ali M Harandi, Bertrand Dubois, Dominique Kaiserlian

**Affiliations:** 1 CIRI, International Center for Infectiology Research, Mucosal Immunity, Vaccination & Biotherapy Laboratory, Inserm U-1111, CNRS UMR5308, Université Claude Bernard Lyon 1, Ecole Normale Superieure de Lyon, Lyon, France; 2 CIRI, International Center for Infectiology Research, Immunobiology of Viral Infections Laboratory, Inserm U-1111, CNRS UMR5308, Universite Claude Bernard Lyon 1, Ecole Normale Superieure de Lyon, Lyon, France; 3 Department of Microbiology and Immunology, Institute of Biomedicine, Sahlgrenska Academy, University of Gothenburg, Gothenburg, Sweden

## Abstract

Development of vaccines able to induce mucosal immunity in the genital and gastrointestinal tracts is a major challenge to counter sexually transmitted pathogens such as HIV-1 and HSV-2. Herein, we showed that intradermal (ID) immunisation with sub-unit vaccine antigens (i.e., HIV-1 gp140 and HSV-2 gD) delivered with Poly(I:C) or CpG1668 as adjuvant induces long-lasting virus-specific immunoglobulin (Ig)-G and IgA antibodies in the vagina and feces. Poly(I:C)-supplemented sub-unit viral vaccines caused minimal skin reactogenicity at variance to those containing CpG1668, promoted a delayed-type hypersensitivity (DTH) to the vaccine and protected mice from genital and neurological symptoms after a lethal vaginal HSV-2 challenge. Interestingly, Poly(I:C_12U_) (Ampligen), a Poly(I:C) structural analogue that binds to TLR3 but not MDA-5, promoted robust mucosal and systemic IgG antibodies, a weak skin DTH to the vaccine but not IgA responses and failed to confer protection against HSV-2 infection. Moreover, Poly(I:C) was far superior to Poly(I:C_12U_) at inducing prompt and robust upregulation of IFNß transcripts in lymph nodes draining the injection site. These data illustrate that ID vaccination with glycoproteins and Poly(I:C) as adjuvant promotes long-lasting mucosal immunity and protection from genital HSV-2 infection, with an acceptable skin reactogenicity profile. The ID route thus appears to be an unexpected inductive site for mucosal immunity and anti-viral protection suitable for sub-unit vaccines. This works further highlights that TLR3/MDA5 agonists such as Poly(I:C) may be valuable adjuvants for ID vaccination against sexually transmitted diseases.

## Introduction

Mucosal immune responses are deemed critical for the prevention of sexually transmitted diseases, such as those induced by human immunodeficiency virus-1 (HIV-1) and herpes simplex virus type 2 (HSV-2). The respective roles of mucosal IgG and IgA antibodies (Abs) for providing protective immunity in the genital tract are not completely understood. Indeed, the presence of neutralising mucosal IgA Abs against HIV-1 envelope glycoproteins is a key correlate of protection in highly exposed uninfected individuals that remain IgG seronegative.^1–3^ A prophylactic vaccine able to generate virus-specific IgG and IgA Abs in the vagina and rectum would thus be of paramount importance to limit heterosexual transmission.

Parenteral routes such as the subcutaneous (s.c.) and the intramuscular routes, classically used for vaccination are notoriously inefficient at generating mucosal IgA responses. Mucosal immunity, especially in the gastrointestinal tract, is best induced by mucosal vaccination and the ‘inductive site’ where initial priming/vaccination occurs conditions the ‘effector site’ where mucosal effector responses will take place.^4^ Much less is known, however, concerning regional specialisation of immunity in the genital tract. Although intravaginal (ivag) and intranasal (IN) immunisations can generate immunity in the genital tract in mice and humans,^5^ each route has limitations for mass vaccination, including acceptability and risk of leakage to the central nervous system. Thus, identification of non mucosal vaccine delivery routes able to confer immunity in the genital tract remains a challenge.

Immunisation through the skin has recently attracted interest as a mean for inducing mucosal IgA Ab responses. Indeed, a pioneering study by Glenn *et al.* documented in mice that transcutaneous immunisation with cholera toxin induces IgA Abs in the lungs and protection against a nasal challenge with cholera.^6^ Moreover, we have previously reported in humans that TC delivery of the live-attenuated measles vaccine ROUVAX using a patch generates a transient salivary IgA Ab response,^7^ supporting the notion that skin might be an inductive site for mucosal Ab responses in humans. Although transcutaneous immunisation mostly targets epidermal Langerhans cells, intradermal (ID) vaccine delivery offers the advantage of targeting both epidermal Langerhans cells and dermal DC, which are highly efficient at inducing T-and B-cell immunity.^8^ However, few vaccines (e.g., BCG, smallpox and rabies) are delivered via the ID route so far. Importantly, ID vaccination allows antigen dose sparing and recent development of new ID injection devices increasing compliance opens the way for wider usage for mass vaccination. We have previously documented the superiority of ID microneedle vaccination against seasonal flu over the classical intramuscular vaccination route in immunocompromised transplanted patients.^9^ More recently, we showed that ID vaccination with viral glycoproteins protected domestic swine against a lethal respiratory infection with a porcine herpes virus, as efficiently as intramuscular vaccination with the gold standard.^10^ Yet, similar to other parenteral routes of immunisation, the ID route seems to be poorly efficient at inducing mucosal Abs, especially IgA, even in the presence of adjuvants such as the double mutant of heat-labile *Escherichia coli* enterotoxin dmLT,^11–13^ the Toll-like receptor ligand (TLR-L)-4 monophosphoryl lipid A^14^ or thymic stromal lymphopoietin.^15^ This highlights the need to identify ID adjuvants suitable to elicit mucosal immunity.

In contrast to traditional vaccines consisting of live-attenuated or killed pathogens, which elicit robust immune responses, but potential pathogenicity and modern vaccines increasingly consist of inert pathogen components, recombinant proteins or glycoproteins to improve safety profile and manufacturing reproducibility. However, such sub-unit vaccines are often poorly immunogenic and require the use of adjuvants to enhance their immunogenicity. TLR ligands are attractive candidate adjuvants for anti-infectious vaccines, since signalling via TLRs stimulates both humoral and cellular responses induced by DCs. For example, harnessing TLR3 and TLR9 ligands, i.e., Poly(I:C) and CpG, was shown to elicit both cellular and humoral immunity to protein antigens.^16–21^ Thus, identification of adjuvants mimicking pathogen-associated molecular patterns that (i) do not induce adverse reactions and hence applicable to humans, (ii) confer the adequate immune response and (iii) target the mucosal site that is most appropriate for protection against pathogen infection, remains a major challenge.

In the present study, we investigated in mice the potential of ID delivery of sub-unit vaccines in combination with an adjuvant to generate specific Ab responses in the genital and intestinal tracts. We show that long-lasting HIV-1 gp140-specific IgG and IgA Ab responses in the vagina can be induced by ID, but not subcutaneous (s.c.), immunisation with trimeric HIV-1 gp140 in the presence of the TLR3 agonist Poly(I:C), with minimal skin reactogenicity. In addition, a similar protocol using the HSV-2 glycoprotein D (gD) conferred protection against a lethal genital infection with HSV-2 and prevented genital and neurological pathology.

## Results

### ID immunisation with trimeric HIV-1 gp140 and selected adjuvants induces mucosal antibody responses

**Figure 1:**
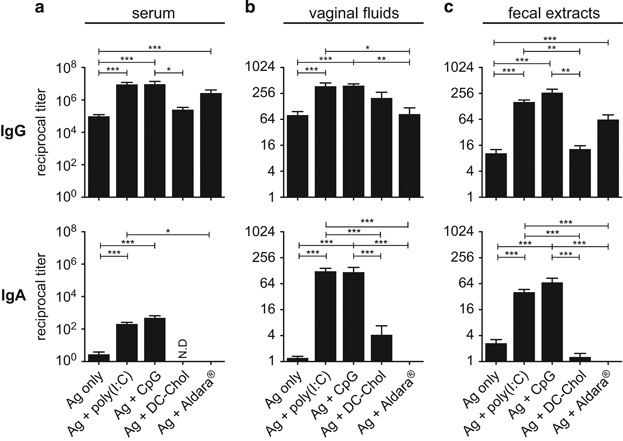
Adjuvants promoting mucosal Abs to HIV-1 gp140 after ID vaccination. HIV-1 gp140 IgG and IgA were titrated at day 28 in serum (**a**), vaginal fluids (**b**) and fecal extracts (**c**) from mice immunised ID with gp140 alone or in the presence of either Poly(I:C), CpG, DC-Chol or Imiquimod (Aldara). Results are expressed as mean+s.e.m. of Ab titers from pooled experiments representing a total of 12 (DC-chol, Aldara), 20 (CpG) and 80 (gp140 alone, gp140+Poly(I:C)) mice. Statistics using the Kruskall–Wallis test and Dunn’s multiple comparisons.

**Figure 2:**
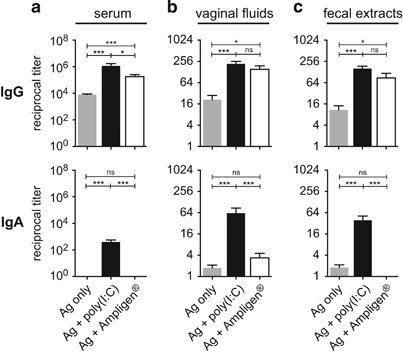
Ampligen promotes HIV-1 gp140-specific mucosal IgG but not IgA. Mice were immunised ID with either gp140 alone or together with Poly(I:C) or Ampligen. On day 28, gp140-specific IgG and IgA were titrated in serum (**a**), vaginal fluids (**b**) and fecal extract (**c**). Data represented mean+s.e.m. of Ab titer from three pooled experiments representing a total of 10–24 mice per group. Statistics using the Kruskal–Wallis test and Dunn’s multiple comparisons.

Several adjuvants were compared for their ability to induce mucosal IgG and IgA Abs to gp140 after ID immunisation. Three ID immunisation with gp140 alone induced high levels of specific IgG Abs in the serum (Figure 1a) and lower levels in vaginal fluids (Figure 1b) and fecal extracts (Figure 1c), but no appreciable levels of specific IgA Abs were detected in either fluids (Figures 1a–c). Comparison of the adjuvant effect of the TLR ligand Poly(I:C), CpG-1668 and Imiquimod (Aldara) and of the cationic lipid DC-Chol, showed that all adjuvants, except DC-Chol, enhanced the level of gp140-specific IgG Abs in serum and both mucosal fluids, after ID co-administration with gp140. Interestingly, only Poly(I:C) and CpG-1668 induced gp140-specific IgA Abs in the serum, vaginal fluids and fecal extracts (Figure 1a–c). Although vaginal IgG Abs were observed after 2 immunisations, induction of IgA Ab production in the genital tract required three immunisations (Supplementary Figure 1), thus all subsequent experiments were carried out using three immunisations. In contrast, the Poly(I:C) analogue Poly(IC_12U_; i.e., Ampligen),^22^ which binds TLR3 but not melanoma differentiation-associated gene 5 (*MDA-5*) and has a shorter half life,^23^ enhanced gp140-specific IgG Ab responses in the sera, vaginal fluids and fecal extracts as efficiently as Poly(I:C), but was poorly efficient at inducing specific IgA Abs in vaginal fluids and fecal extracts (Figure 2). Thus, although CpG1668, Poly(I:C) and Ampligen exhibited comparable ability to stimulate mucosal and systemic IgG Ab responses, the highest specific mucosal IgA responses were induced by Poly(I:C) and CpG1668.

### ID delivery of gp140 with Poly(I:C) allows for priming of delayed-type hypersensitivity without overt skin reactogenicity

**Figure 3:**
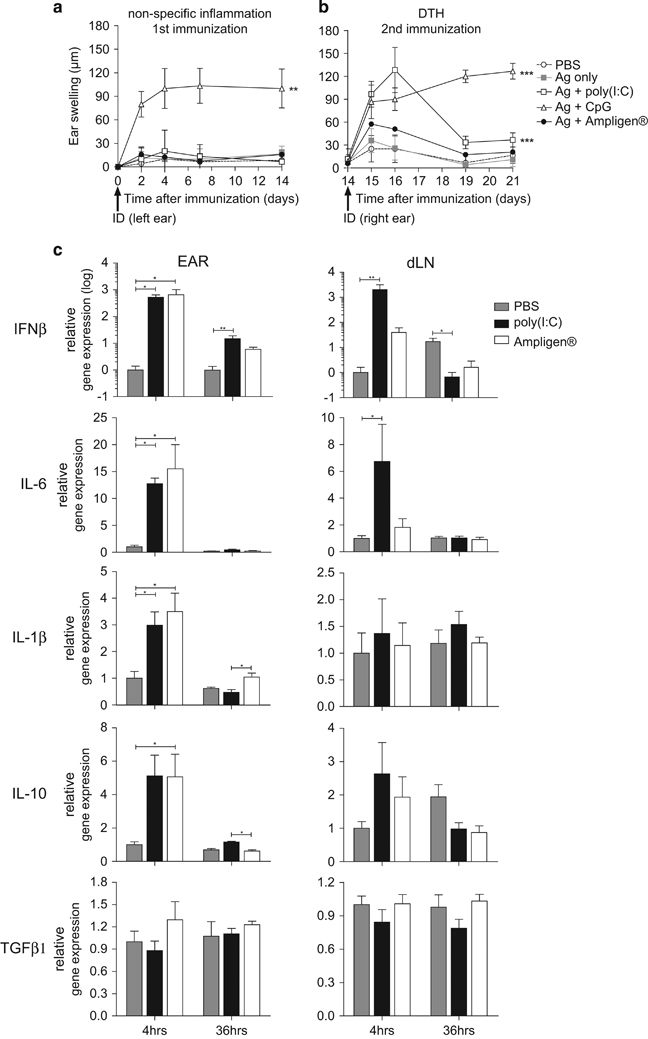
ID delivery of Poly(I:C) triggers skin and lymph node innate immunity and allows for priming of a DTH response without overt skin reactogenicity. Non-specific skin inflammation was assessed by ear swelling at various time points after the first ID injection of gp140 alone or together with either Poly(I:C), CpG or Ampligen (**a**). The vaccine specific DTH response was determined by ear swelling at various days after the second ID injection performed on day 14. Results are expressed as mean+s.e.m. of ear swelling (μm) in 6 mice per group and are representative of one out of two experiments (**b**). Statistical analyses were performed using the two-way analysis of variance (ANOVA) test and Bonferroni’s multiple comparisons. Asterisks indicate significance compared with the PBS control group. Quantitative PCR analysis of cytokine transcripts were carried out at 4 and 36 h after ID injection of poly(I:C), Ampligen or PBS as control. Results from four individual mice expressed as mean±s.e.m. of cytokine messenger RNA (mRNA) ratios to housekeeping gene and normalised to PBS 4 h are shown (**c**). Statistical analyses were performed using Kruskall–Wallis test with Dunn’s Multiple comparison.

Skin reactogenicity (i.e., non-specific inflammation), as determined by the ear-swelling response that developed after the first ID immunisation, was virtually undetectable up to 14 days after injection of either phosphate-buffered saline (PBS), gp140 alone or together with Poly(I:C) or Ampligen (Figure 3a). In contrast, ID injection of gp140 with CpG-1668 as adjuvant induced non-specific inflammation peaking at day 2 that was sustained up to day 14 (Figure 3a). The delayed type hypersensitivity (DTH) response was determined by the ear swelling in response to the second immunisation performed on the contralateral ear at day 14. As shown in Figure 3b, gp140 plus Poly(I:C) generated a classical DTH response that peaked at 48 h and progressively resolved within 3 days, although a weak DTH response was observed when Ampligen was used as adjuvant. Alternatively, gp140 plus CpG caused a massive and sustained ear swelling, most likely resulting from the combined effects of non-specific skin inflammation and of the DTH response. Thus, when co-administrated ID with gp140, Poly(I:C)-induced limited skin reactogenicity, contrary to CpG1668, and promoted a DTH response to the vaccine reflecting priming of vaccine-specific T cells. Given the unacceptable skin reactogenicity induced by CpG 1668, the rest of the study was performed with Poly(I:C) and Ampligen.

### Innate immune signals induced by ID injection of Poly(I:C) versus Ampligen

We next examined whether ID immunisation triggered innate immune signals relevant to the IgA response. Messenger RNA transcripts of cytokines associated with innate immunity (IFNß, IL-1ß) or involved in IgA B cell switch and plasma cell differentiation (IL-10, TGFß, IL-6) were analysed in the ear (i.e., the ID injection site) and draining cervicomandibular LN (dLN) at 4 and 36 h after injection of either Poly(I:C), Ampligen or PBS as control. In the skin, Poly(I:C) and Ampligen similarly up-regulated messenger RNA transcripts of interferon (IFN)-ß and to a minor extend of interleukin (IL)-6, IL-1ß and IL-10, while TGFß transcripts were not upregulated (Figure 3c). In contrast, in dLN, poly(I:C) was by far superior to Ampligen to trigger high transcription of IFN-ß and to a minor extend of IL-6, while both adjuvant poorly stimulated IL1ß and IL10 and failed to enhance TGFß transcripts. These data thus showed that Poly(I:C), at variance to Ampligen. has a unique efficacy to promptly and transiently trigger a robust IFNß response and to upregulate IL-6 transcripts in skin dLN after ID injection.

### ID immunisation generates specific IgG and IgA Ab-secreting cells in uterus and intestine

**Figure 4:**
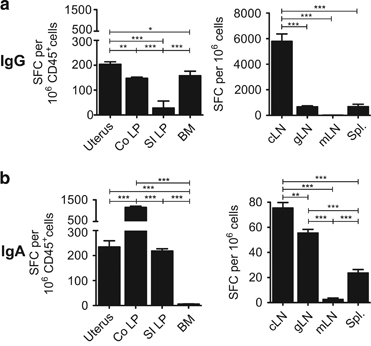
Ab-producing cells induced by ID immunisation with gp140+Poly(I:C). IgG (**a**) and IgA (**b**) SFC were measured by ELISPOT assay 5 days after the last ID immunisation with gp140+Poly(I:C) in uterus, colon, small intestine and bone marrow (left panels) and in cLN, mLN, gLN and spleen (right panels). Data are expressed as mean+s.e.m. of gp140-specific SFC/10^6^ total (*right panels*) or CD45^+^ cells (*left panels*) pooled from 6–8 mice /group. Statsitical analyses were performed using one-way analysis of variance (ANOVA) with Tukey’s multiple comparison test.

To determine whether mucosal Ab responses after ID immunisation with gp140 and Poly(I:C) resulted from local Ab secretion by mucosal Ab-producing cells, gp140-specific spot-forming cells (SFCs) were analysed 5 days after the third ID immunisation. Numerous gp140-specific IgG SFC were detected in uterus, colon and bone marrow, and much less in the small intestine (Figure 4a, left). Specific IgA SFC were present at similar frequencies in the uterus and small intestinal lamina propria and greatly increased in the colon, whereas virtually undetectable in the bone marrow (Figure 4b, left). Analysis of lymphoid organs showed that cervical LN (cLN) contained the highest number of gp140-specific IgG SFC, as compared with genital LN (gLN) and spleen, whereas no response was detected in mesenteric LN (mLN; Figure 4a, right). Likewise gp140-specific IgA SFC were the highest in cLN and gLN, low in spleen and virtually undetectable in mLN (Figure 4b right). Along these lines, gp140-specific IgA Ab could be detected in culture supernatant of cLN, but not mLN, cells after the second ID immunisation (Supplementary Figure 3), suggesting that the IgA Ab response was unlikely primed in mLN.

These data indicate that ID immunisation with gp140 plus Poly(I:C) efficiently generates gp140-specific Ab-producing cells in the genital and gastrointestinal mucosae, which likely contribute to the specific IgG and IgA Ab responses detected in the vaginal fluids and fecal extracts.

### ID, but not s.c., immunisation with gp140 and Poly(I:C) induces long-lasting mucosal and systemic Ab responses

**Figure 5:**
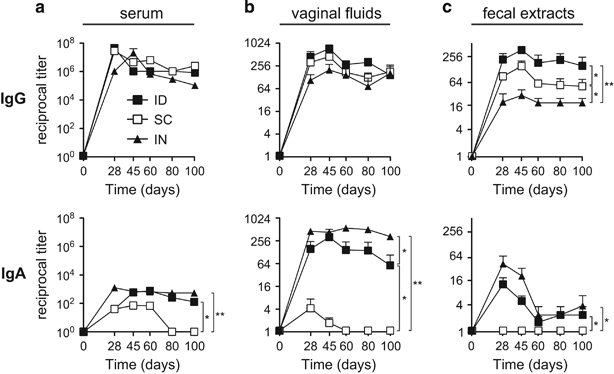
HIV-1 gp140-specific IgG and IgA responses induced via ID route compared with those induced via s.c. and IN routes. Gp140-specific IgG (top panels) and IgA (bottom panels) were titrated in serum (**a**), vaginal fluids (**b**) and fecal extracts (**c**) of mice at various time points after the last immunisation with gp140 plus Poly(I:C) via either the ID (black squares), s.c. (white squares) or IN (black triangles) route. Results are expressed as mean+s.e.m. of Ab titres and are representative of one out of two experiments using 6 mice per group. Statistical analyses were performed using the two-way analysis of variance (ANOVA) test and Bonferroni’s multiple comparisons.

We next examined the duration of mucosal gp140-specific antibody responses generated by ID immunisation with Poly(I:C) as adjuvant. The s.c. route, which by-passes the skin and is poorly efficient at priming mucosal immunity, was used as a negative control to assess whether mucosal IgA induction results from the sole use of poly(I:C) or also requires delivery through the ID route. The IN route, known for its ability to induce high levels of vaginal IgA Abs,^5^ was used as a positive control. Although all three immunisation routes generated comparably high levels of gp140-specific IgG Abs in serum (Figure 5a) and vaginal fluid (Figure 5b) up to 100 days after immunisation, the ID and s.c. routes were more efficient than the IN route to induce mucosal IgG Ab responses, especially in feces (Figure 5c). Remarkably, mucosal IgA responses in the vaginal fluid and fecal extracts were observed only after IN and ID, but not s.c., immunisation with gp140 plus Poly(I:C) (Figure 5b,c, bottom panels). In particular, ID immunisation elicited a robust and long-lasting gp140-specific vaginal IgA Ab response, at levels slightly lower than those induced by IN immunisation (Figure 5b bottom panel). Thus, whereas systemic and vaginal IgG can be induced equally well irrespective of the immunisation route, the ID, but not the s.c., route allows for induction of a specific and long-lasting IgA response in the vagina indicating, that Poly(I:C) and a dedicated immunisation route are both required to induce IgA Ab.

### ID vaccination with Poly(I:C) and HSV-2 gD protects against genital HSV-2 infection

**Figure 6:**
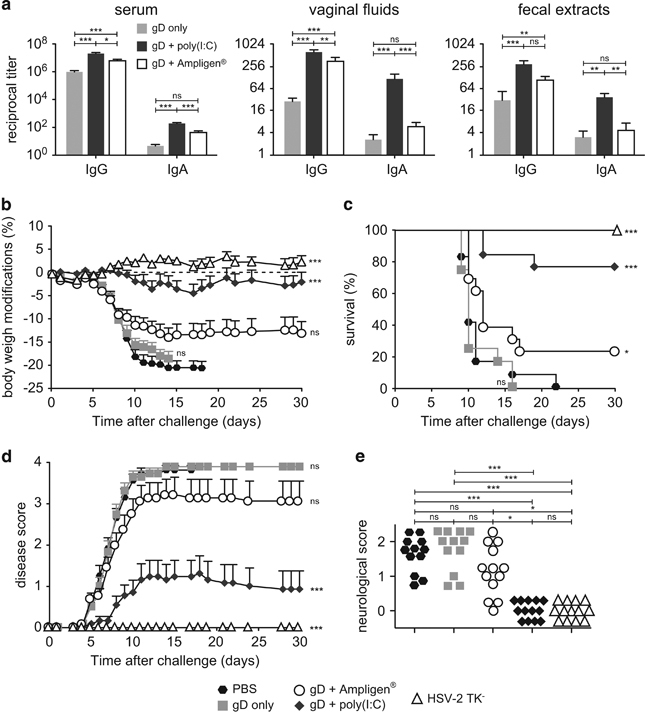
ID vaccination with HSV-2 gD glycoprotein with Poly(I:C) induces mucosal and systemic immunity and protects from lethal vaginal HSV-2 infection. (**a**) Mice were immunised ID with HSV-2 gD alone or together with Poly(I:C) or Ampligen and gD-specific IgG and IgA were measured 7 days after the last immunisation, in serum, vaginal fluids and fecal extracts. Data represent mean+s.e.m. of Ab titres of six experiments each using 6–7 mice per group. (**b**–**e**) Mice were injected three times ID with either PBS, gD alone, gD+Poly(I:C), gD+Ampligen or once ivag with HSV-2 tk^−^. One week after the last immunisation, mice were challenged ivag with 10^4^ plaque-forming units (PFU) of virulent HSV-2 and protection against infection was followed by body weight change (**b**), survival (**c**), clinical score (**d**) and neurological score (**e**). Data correspond to a pool of two experiments (**b**–**e**) each with 6–7 mice per group. Statistical analyses were performed using the Kruskall Wallis test and Dunn’s multiple comparisons (**a**,**e**), the two-way analysis of variance (ANOVA) test and Bonferroni’s multiple comparisons (**b**,**d**) and the Gehan–Breslow–Wilcoxon test (**c**). Asterisks (**b**–**d**) indicate significance compared with the PBS control group.

We next assessed whether ID immunisation with a subunit vaccine adjuvanted with Poly(I:C) could confer anti-viral mucosal and systemic protection. Because there is no model of HIV infection in mice, we used a well-established mouse model of genital infection with HSV-2, a virus close to HIV, which is also transmittable via the female genital tract. Immunisation with HSV-2 gD protein alone induced high levels of anti-gD IgG in serum, lower levels of IgG in the vagina, but barely detectable IgA. As observed with HIV-1 gp140, vaginal gD-specific IgA Ab response was much higher with Poly(I:C) than Ampligen, although both adjuvants augmented the specific IgG Ab response in vaginal fluids, fecal extracts and sera (Figure 6a). ID immunisation with gD plus Poly(I:C) or Ampligen did not give rise to skin reactogenicity (Supplementary Figure 2A) and a skin DTH response to the vaccine was observed with Poly(I:C) but not Ampligen (Supplementary Figure 2B). Following HSV-2 genital challenge, mice injected with PBS or gD alone developed a rapidly progressing disease characterised by ~20% body weight loss, severe and purulent genital lesions and hind limb paralysis (Figures 6b–e). In contrast, similarly to mice immunised ivag with HSV-2 tk^−^ and mice vaccinated ID with gD+Poly(I:C) were protected from disease, as shown by virtual absence of body weight loss (Figure 6b), 80% survival up to 1 month after challenge (Figure 6c) and strongly decreased disease score (Figure 6d). Alternatively, mice vaccinated with gD+Ampligen rapidly developed severe pathogenesis, similar to mice immunised with gD alone resulting in 70% mortality from infection (Figure 6b–d). Further detailed monitoring of the severity of neurological symptoms along with genital scores (Figure 6e) revealed that all mice of PBS and gD alone-vaccinated groups exhibited severe genital lesions (score 3) and neurological symptoms starting from uncoordinated body movements and muscular leg weakness (score 1) and rapidly leading to hind limb paralysis (score 2). Similarly to the HSV-2 tk^−^ vaccinated mice, none of the mice vaccinated with gD+Poly(I:C) developed neurological symptoms, whereas 10/13 mice immunised with gD+Ampligen did so. These data underscore that ID vaccination with Poly(I:C) as adjuvant is efficient to generate mucosal and systemic IgG and IgA Ab responses and to confer protection from genital HSV-2 infection.

## Discussion

Vaccine against sexually transmitted diseases, such as those induced by HIV-1 and HSV-2 infections, should ideally confer protection in the genital tract and rectum. Such protective mucosal immunity is likely to be generated more efficiently after immunisation via mucosal routes rather than systemic routes, which are classically used for vaccine delivery. Nevertheless, development of mucosal vaccines for several life-threatening pathogens has been hampered by the lack of potent mucosal adjuvants devoid of overt toxicity. Development of alternative (systemic) routes able to confer both systemic and mucosal immunity and protection at the mucosal tissues remains a challenge. In this study, we show that ID vaccination with a sub-unit vaccine and Poly(I:C) as adjuvant induces mucosal IgG and IgA Ab responses both in the genital and gastrointestinal tracts and confers protection against a lethal vaginal viral infection.

We could show herein that ID immunisation with HIV-1 gp140 alone was sufficient to induce specific IgG in the serum, vaginal fluids and fecal extracts, but that induction of mucosal IgA responses required co-administration of an adjuvant and three immunisations. Of the various adjuvants tested, only Poly(I:C) and CpG1668 had the ability to induce IgA Ab responses in the vaginal fluids and fecal extracts and both enhanced systemic and mucosal gp140-specific IgG responses. Intriguingly, mucosal IgA-producing cells either largely exceeded mucosal IgG-producing cells (as observed in the colon LP) or were found in comparable numbers (as seen for uterus), while IgG titres always overrode those of IgA in vaginal and fecal fluids. This is most likely explained by the fact that mucosal IgG levels are accounted for by both local production by mucosal plasma cells and plasma transudation, whereas IgA may essentially originate from mucosal plasma cells. Poly(I:C) caused minimal non-specific skin inflammation at the site of injection, (contrary to CpG 1668), and elicited long-lasting gp140-specific IgG and IgA production in mucosal fluids and a vaccine-specific DTH indicating priming of a vaccine-specific effector T-cell response. The magnitude and duration of gp140-specific vaginal IgG and IgA Ab responses induced by ID immunisation were comparable to those induced by IN immunisation, i.e., the gold standard route for vaginal IgA responses. By contrast, s.c. immunisation with gp140+Poly(I:C), which by-passes the skin, was poorly efficient at inducing a vaginal IgA Ab response. Thus, the site of antigen delivery and the nature of the adjuvant both critically condition induction of mucosal IgA Ab responses, and combining Poly(I:C) with ID immunisation appears highly efficient in this respect.

Our study highlights that induction of mucosal IgA Ab responses after ID immunisation requires the use of dedicated adjuvants (e.g., CpG and Poly(I:C)). The reason why Ampligen, a structural Poly(I:C) analogue with immune-stimulatory properties,^22,24^ potentiated vaccine-specific mucosal and systemic IgG Ab responses, but was unable to promote mucosal specific IgA Ab responses when delivered ID either HIV gp140 or HSV-2 gD, remains to be elucidated. At variance to Poly(I:C), Ampligen signals via TLR3 but not MDA-5 and is a less potent inducer of type-I IFN.^23^ Along these lines, huge levels of IFN-ß transcripts were rapidly induced in skin dLN by Poly(I:C), but not by Ampligen. As type-I IFN strongly promotes humoral responses including IgA in both human^25^ and mice,^26^ it could be hypothesised that the unique ability of Poly(I:C) over Ampligen to induce mucosal IgA responses might result from its far superior ability to upregulate IFNß in skin dLN. Thus, dual capacity of induction of mucosal IgA responses and protection against viral infection should be taken into consideration when selecting detoxified TLR3 agonist as safe anti-infectious vaccine adjuvants for human use.^27^

Several plausible mechanisms may account for the mucosal B-cell responses induced after ID immunisation using Poly(I:C) as adjuvant, including licensing of dendritic cells for B-cell IgA isotype switching, induction of mucosal homing receptor expression and migration of B cells or plasmablasts, and induction of IgA B-cell/plasmablasts survival and differentiation factors. Although the cellular and molecular mechanisms supporting IgA Ab production in distant mucosal tissues after ID immunisation remain unknown, our data indicate that they differ from those involved in transcutaneous immunisation. Indeed, it has been proposed that after transcutaneous immunisation, mucosal immunity (IgA) is primed in Peyer’s patches and mLN by DCs either migrating from the immunisation site^28^ or rapidly differentiating *in situ* from bone marrow precursors.^29^ In contrast, our data suggest that the IgA B cell response after ID immunisation is mainly induced both in skin-draining cLN and distant gLN (but not mLN), since gp140-specific IgA-producing cells were present in these lymphoid organs, besides mucosal tissues. That the IgA Ab response was not primed in mLN is further supported by the detection of gp140-specific IgA in culture supernatant of cLN, but not mLN, cells after the second ID immunisation (Supplementary Figure 3). These observations support that IgA isotype switching and IgA plasma cell differentiation after ID immunisation are induced primarily in cLN and gLN rather than in the gut-associated mLN. Whether cLN and gLN have complementary roles in the induction of IgA plasma cells aimed to migrate to mucosal tissues or prime different populations with specific homing potential remains to be determined. Priming of the IgA Ab response in the distant gLN after ID immunisation might require migration of DC from the immunisation site, as recently documented for CD8^+^ T-cell cross-priming after sublingual immunisation.^30^ The nature of the skin DC subset responsible for mucosal Ab production after ID immunisation remains to be explored. However, the human dermal CD14^+^ DC subset, which contrary to Langerhans cells and CD1a^+^ dermal DCs efficiently induce IgA B-cell differentiation,^31^ and the mouse Langerin^+^ DCs^29^ are likely candidates.

Mucosal imprinting of IgA-producing cells after ID immunisation might involve induction of the chemokine receptor CCR10, which promotes plasma cell migration to both the colon^32^ and the genital tract.^33^ CCR10-dependent homing of IgA plasma cells into the genital tract has been demonstrated after IN immunisation^34^ and expression of CCR10 can be induced on terminally differentiated B cells by Vitamin D3 metabolites produced in the skin.^35^ Moreover, the two adjuvants that best induce mucosal IgA Ab responses through the ID route, i.e., Poly(I:C) and CpG, have been shown to promote mucosal homing. Indeed, distant delivery of CpG upregulates expression of the CCR10 ligand CCL28 in the uterine epithelium,^34^ and Poly(I:C) induces gut homing receptors on lymphocytes.^36^ We were nevertheless unable to pinpoint the impact of ID vaccination on mucosal homing receptor expression on IgA-switched B cells and plasmablasts in the skin-draining LNs owing to the very low number of IgA-committed B cells and plasmablasts retrieved from the draining LNs.

Importantly, we demonstrated that prophylactic ID vaccination with a sub-unit vaccine confers protection from a genital viral infection. Indeed, ID vaccination with recombinant gD of HSV-2 and Poly(I:C) protected mice against a lethal genital challenge with a virulent HSV-2 strain, nearly as efficiently as the gold standard ivag immunisation with the HSV-2 tk^−^ mutant virus. Vaccination with live attenuated HSV-2 tk^−^ virus. The latter was used as a positive control, owing to its ability to confer full protection against genital HSV-2 infection in mice.^37^ However, the use of live attenuated HSV-2 in human is hampered by potential safety concerns including risk of reversion to virulence and spread to central nervous system. Current research on vaccine against genital herpes is geared towards sub-unit HSV-2 vaccines, yet still no vaccine has proven to provide immune protection in field studies. Our finding that ID vaccination with HSV-2 gD and Poly(I:C) confers high level of protection (nearly 80%) in mice against both genital lesions, body weight loss and death due to neurological symptoms therefore warrants further exploration. It is possible that combination of several viral antigens from HSV-2 may confer higher degree of protection ideally similar to that provided by HSV-2 tk^−^. This may pave the way for further human vaccination strategies against genital herpes.

Remarkably, mice vaccinated with gD and Ampligen, which displayed high titres of gD-specific IgG Ab in their vagina, but a low specific vaginal IgA response, were poorly protected from genital infection. This may reflect a critical role of the gD-specific genital IgA antibodies, although the contribution of virus-specific T cells in anti-viral protection could not be ruled out as Ampligen has a lower ability to induce vaccine-specific DTH response than Poly(I:C). More likely, because Poly(I:C) promotes cross-priming of cytotoxic CD8^+^ T cells,^38^ both virus-specific T cells and mucosal Abs might act in concert following ID vaccination for protection against a viral vaginal infection.^39^

Altogether, our data underscore that specific mucosal IgG and IgA responses and protection against genital viral infection can be achieved by ID vaccination with a sub-unit vaccine and Poly(I:C) as adjuvant, without overt skin reactogenicity. Besides, through provision of specific IgA in the vaginal fluids, this vaccination approach might protect from heterosexual viral transmission. It should, however, be noted that ID vaccination into the mouse ear may not directly translate to human ID vaccination, as the site used in human is different (i.e., the deltoid). While (hairless) mouse and human skin appear to share many common features with respect to micro-anatomy and microvascularisation,^40^ there are certainly marked differences between these two species with respect to anatomic distribution of cells and vessels. Thus, one should be cautious before generalising our findings and hence future clinical testing in human is warranted. Considering that new ID micro-needle devices are now suitable for mass vaccination without the need of trained personnel, ID vaccination with subunit vaccines and TLR3/MDA5 agonists such as Poly(I:C), might be a promising approach to protect from sexually transmitted diseases and especially HSV2 infection, which is still in need for a protective sub-unit vaccine.^41^

## Materials and methods

### Mice

Female BALB/c mice were purchased from Charles River Laboratories (L'Arbresle, France) and used at 7–10 week of age. All experiments were previously approved by the local Ethic committee (CECCAPP Lyon, registered by the French National Ethics Committee of Animal Experimentation under no. 311 and no. ENS 2013 006) and were performed at PBES (Lyon) in accordance with the European guidelines for animal experiments.

### Antigens

Trimeric recombinant gp140 (gp120 plus the external domain of gp41) envelope protein from the HIV-1 Clade A strain UG37 was produced as a recombinant product in CHO cells and obtained from Polymun Scientific (Vienna, Austria). Recombinant histidine-tagged gD from HSV-2, consisting of the extracellular portion of gD (AA 1–142) from the HSV-2 strain 333, was prepared in CHO cells as previously described.^42^

### Immunisations

All mice were injected s.c. with 2 mg Depo-Promone (Pharmacia SAS, Guyancourt, France) 5 days before the first immunisation to allow synchronisation of the oestrous cycle for IgA monitoring in their vaginal fluids.^5^ Mice were immunised on days 0, 14 and 21 by ID injection into the ear pinnae (left, right and left, respectively) of 10 μg HIV-1 gp140 or HSV-2 gD alone in PBS or mixed with adjuvant, in a final volume of 15 μl. The dose of 10 μg Ag was selected after preliminary dose–response (5, 10 and 20 μg) and appeared as the optimal dose able to reproducibly induce an IgA response. The ear pinnae was selected as the site of ID injection to provide direct and accurate delivery of a desired quantity of Ag into the dermis, without leakage to the s.c. space (absent in the ear). Alternatively, mice received 10 μg of gp140 with or without adjuvant in 10 μl (5 μl per nostril) for IN immunisation and in 100 μl for s.c. immunisation at the base of the neck. Adjuvant used were: Poly(I:C) (25 μg; InVivoGen, San Diego, CA, USA), Poly(I:C_-12U_) i.e., Ampligen (25 μg; Hemispherx, Biopharma, Philadelphia, PA, USA) was a kind gift from L. Zitvogel (Institut Gustave Roussy, Villejuif, France); CpG-B (10 μg ODN 1668, 5′- TCCATGACGTTCCTGATGCT-3′(20mer), InVivoGen), the cationic lipid 3β-(*N*-(*N*′, *N*′-dimethylaminoethane) carbamoyl cholesterol (DC-Chol, 50 μg)^43^ and Aldara cream 5% (Imiquimod, a kind gift from Christophe Caux, CRCL, Lyon, France), a TLR7 ligand administered by ear painting (10 μl) before each immunisation.

### Skin reactogenicity and DTH response

Skin reactogenicity and DTH responses were tested as described.^44^ Ear swelling was measured using calipers, at various times after the first ID immunisation performed on the left ear. Skin DTH response was determined by measure of ear swelling after the second ID immunisation (right ear). Ear swelling (μm) was calculated as the difference between the ear thickness before and after immunisation as described.^44^

### Sample collection

Vaginal fluids were collected by four successive washes of the vaginal cavity with 50 μl of PBS supplemented with a cocktail of protease inhibitors (Complete, Roche Diagnostics GmbH, Mannheim, Germany). Fresh fecal pellets were collected in PBS plus protease inhibitor (400 μl per 100 mg of feces), crushed, centrifuged and supernatants (fecal fluids) were immediately stored at −20 °C until use.

### ELISA titration of antigen-specific IgG and IgA Abs

Briefly, 96-well Maxisorp microtitration plates (Nunc, Roskild, Danmark) were coated with 1 μg/ml (HIV-1 gp140) or 3 μg/ml (HSV-2 gD) of antigens overnight at 4 °C in 0.05 mol/l carbonate buffer, pH 9.6 and washed with PBS-0.05% Tween 20 (PBS-T). After 2 h saturation with PBS containing 2% bovine serum albumin, serial dilutions of samples were added and plates were incubated for 2 h at room temperature. After washing with PBS-T, horseradish peroxidase-conjugated anti-mouse IgG or IgA Abs (SouthernBiotech, Birmingham, AL, USA) were then added for 2 h at room temperature and the reaction was developed with 3,3′-5,5′-tétra-methylbenzidine (SureBlue TMB Microwell peroxidase Substrate 1-Component, KPL, Gaithersburg, MD, USA). After 15 min, the reaction was stopped by adding 12,5% H_2_SO_4_ and optical density was read at 450 nm (VersaMax Microplate Reader, Molecular Devices, Wokingham, Berkshire, UK). Specific Ab titers were determined as the inverse of the highest sample dilution giving an optical density at least twofold higher than that of a naïve sample at the same dilution.

### Quantitative real-time RT-PCR

Cervical lymph nodes (cLN) and ears were homogenised with 1 ml of TRIzol reagent (Invitrogen Life Technologies, Carsbad, CA, USA), and total RNAs were isolated according to the manufacturer’s protocol. Complementary DNA were synthesised by extension of a mix of oligo(dT) and random primers with M-MLV reverse transcriptase (all from Invitrogen Life Technologies) in a mixture containing 1 μg of total RNA first digested by RNase-free DNase (2 U/μg RNA). Specific primer sets were designed using BEACON Designer software and were purchased from Invitrogen Life Technologies. The primer sequences (forward/reverse) used were: mIL-1ß, 5′- gcttgtgctctgcttgtgag-3′/5′- cccaagcaatacccaaagaa-3′; mIL-6, 5′- gaggataccactcccaacagacc-3′/5′ aagtgcatcatcgttgttcataca-3′; mIL-10, 5′- ggcgctgtcatcgatttctc-3′/5′ gacaccttggtcttggagcttattaa-3′; mIFN-ß1, 5′- CTGGCTTCCATCATGAACAA-3′/5′- AGAGGGCTGTGGTGGAGAA-3′; mTGF-ß1, 5′- tgacgtcactggagttgtacgg-3′/5′- ggttcatgtcatggatggtgc-3′; mHPRT, 5′- tcattatgccgaggatttgga-3′/5′- cagtggccttccgtgttc-3′; mG3PDH, 5′- gcatggccttccgtgttc-3′/5′- tgtcatcatacttggcaggtttct-3′.

Real-time PCR was performed on an Stratagene MX3000 using the Platinum SYBR Green qPCR Supermix UDG with a Rox Kit (Invitrogen Life Technologies) according to the manufacturer’s instructions. The relative quantity of each transcript was normalised according to the mean of the expression of two different housekeeping genes: hypoxanthine guanine phospho-ribosyltransferase (HPRT) and glyceraldehyde 3-phosphate dehydrogenase (G3PDH).

### Cell isolation

Single-cell suspensions from spleen, cLN, genital (gLN) and mesenteric (mLN) lymph nodes and bone marrow were obtained by mechanical dissociation in RPMI medium (Gibco, Life Technologies, New York, NY, USA) containing 2% fetal calf serum (Lonza Verviers SPRL, Belgium) and 0,1 mg/ml DNAse I (Roche, Basel, Switzerland). For mononuclear cell isolation uterus were cut into small pieces and incubated for 10 min in PBS containing 1 mmol/l DL-Dithiothreitol (DTT, Sigma Aldrich, St Louis, MO, USA) prior to enzymatic digestion for 1 h at 37 °C with 0,1 mg/ml of DNAse I and 1 mg/ml of collagenase/dispase (Roche, Basel, Switzerland) in RPMI/2% fetal calf serum. Leucocytes were enriched to 95–97% by positive selection with PE-conjugated anti-CD45 mAb (BD Pharmingen, San Diego, CA, USA) and anti-PE microbeads (Miltenyi Biotec) using LS columns (Miltenyi Biotec, Paris, France). Leucocytes from the lamina propria (LP) of small and large intestine were obtained as described elsewhere,^45^ with some modifications. Briefly, tissues were rinsed in PBS/1 mmol/l DTT to remove mucus, cut into pieces and incubated twice for 20 min at 37 °C in PBS/5% fetal calf serum/5 mmol/l EDTA/ 1 mmmol/l DTT to remove epithelial cells and finally incubated for 1 h at 37 °C in RPMI with 12.5 μg/ml Liberase (Roche) and 0.1 mg/ml DNAse. Cells were layered onto a 40–80% Percoll (GE Healthcare, Aulnay-Sous-Bois, France) density gradient and leucocyte collected at the interface were enriched in CD45+ cells by 70–80% and 30–50% for the small and large intestine, respectively.

### B-cell ELISPOT

MultiScreen IP 96-well filter plate (Merck-Millipore, Molsheim, France) were coated overnight at +4 °C with 10 μg/ml gp140 and serial dilution of cells from various tissues were added in duplicate and incubated overnight at 37 °C in 5% CO_2_. Alkaline phosphatase-conjugated goat anti-mouse IgG or IgA Abs (Southern Biotechnology) (1/400 dilution) were added and spots were developed using BCIP/NBT substrate (Sigma Aldrich). SFCs were counted under a light microscope.

### Genital infection with HSV-2

Mice were injected s.c. with Depo-Promone 5 days before ivag challenge with 10^4^ plaque-forming units of the HSV-2 strain 333 administered in 10 μl to mice under Ketamine 50 (17.5 mg/ml)/Xylazine (2.25 mg/ml) anaesthesia. Mice were kept on their back for 30 min to allow infection. Mice were daily examined for body weight, clinical score and survival. Disease severity (including genital and neurological symptoms) was scored as follows: healthy (0); genital erythema (1); swelling and erythema of external vagina (2); severe and purulent genital lesions (3); hind limb paralysis (4). Animals were killed at score 4. A neurological score was also calculated: absence of neuromuscular abnormalities (0); difficulty to walk, uncontrolled body movements and general muscular weakness characterised by difficulty to hang on and/or push with legs (score 1) and end point of the experiment with hind limb paralysis (score 2).

### Statistics

Unless otherwise stated in the figure legends, the following statistical analyses were performed. The non-parametric Kruskal–Wallis test was used to compare three or more experimental groups and was combined with a Dunn’s multiple comparison test to generate *P* values for selected pair wise comparisons. The two-way analysis of variance test was used for kinetics experiments and was combined with a Bonferroni’s multiple comparisons test. Survival data was analysed using the Kaplan–Meier method, and when significant differences among groups were revealed, pairwise comparisons were subsequently analysed by the Gehan–Breslow–Wilcoxon test. All analyses were performed using the GraphPad Prism 4.0 software (San Diego, CA, USA) and *P* values <0.05 were considered significant (*P<0.05; **P<0.01; ***P<0.001).
