# Supplementary material for: Intradermal immunisation using the TLR3-ligand Poly (I:C) as adjuvant induces mucosal antibody responses and protects against genital HSV-2 infection
Source: NPJ Vaccines. 2016 Aug 25;1:16010–. doi: 10.1038/npjvaccines.2016.10 (PMC5707913; doi:10.1038/npjvaccines.2016.10)
Supplement: Supplementary Figure 1 [file npjvaccines201610-s1.pdf]

Supplemental Figure 1. E. Bardel *et al*.

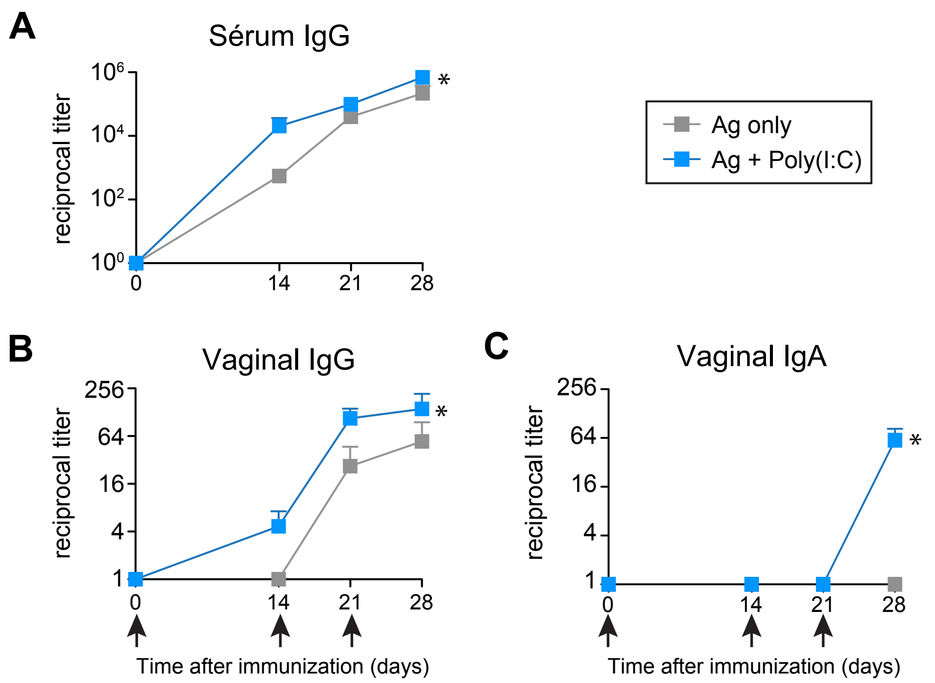

Supplemental Figure 1.

Induction of HIV gp140-specific IgA antibodies requires 3 ID immunizations. gp140-specific serum IgG (A), vaginal IgG (B) and vaginal IgA (C) were titrated by ELISA in blood and vaginal lavages at days 0, 14, 21 and 28 in mice that were immunized ID with gp140 alone (grey) or gp140 + Poly(I:C) (blue) on days 0, 14 and 21. Results are expressed as mean titer + SEM of Ab titer and are from 1 experiment using 6 mice/group. Statistics were performed using 2 way ANOVA.
