# Supplementary material for: Intradermal immunisation using the TLR3-ligand Poly (I:C) as adjuvant induces mucosal antibody responses and protects against genital HSV-2 infection
Source: NPJ Vaccines. 2016 Aug 25;1:16010–. doi: 10.1038/npjvaccines.2016.10 (PMC5707913; doi:10.1038/npjvaccines.2016.10)
Supplement: Supplementary Figure 2 [file npjvaccines201610-s2.pdf]

Supplemental Figure 3. E. Bardel *et al*.

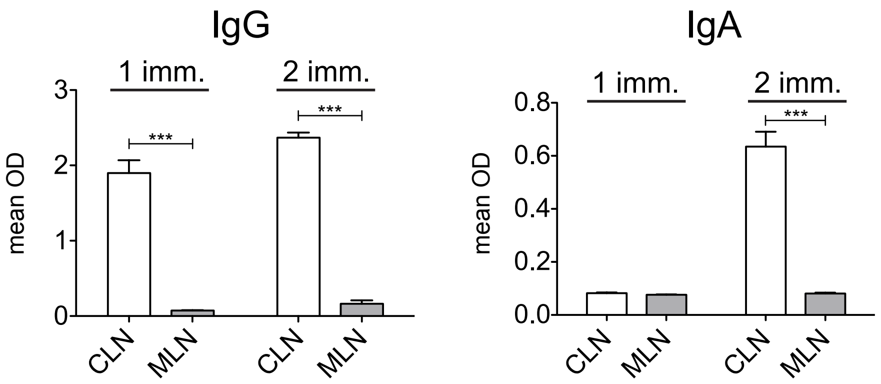

Supplemental Figure 3.

HIV gp140-specific IgA antibodies can be detected in cLN cells supernatants after the second ID immunization. Mice were immunized once or twice (2 weeks interval) with gp140+Poly(I:C) and gD-specific IgG and IgA were titrated in 3 day culture supernatants of cLN and mLN cells (10^6/well) cultured without stimulation in vitro. Results are expressed as mean + SEM and derived from 6 determinations. Statistics were performed using unpaired *t* test with Welch's correction.
