# Supplementary material for: Intradermal immunisation using the TLR3-ligand Poly (I:C) as adjuvant induces mucosal antibody responses and protects against genital HSV-2 infection
Source: NPJ Vaccines. 2016 Aug 25;1:16010–. doi: 10.1038/npjvaccines.2016.10 (PMC5707913; doi:10.1038/npjvaccines.2016.10)
Supplement: Supplementary Figure 3 [file npjvaccines201610-s3.pdf]

Supplemental Figure 2. E. Bardel *et al*.

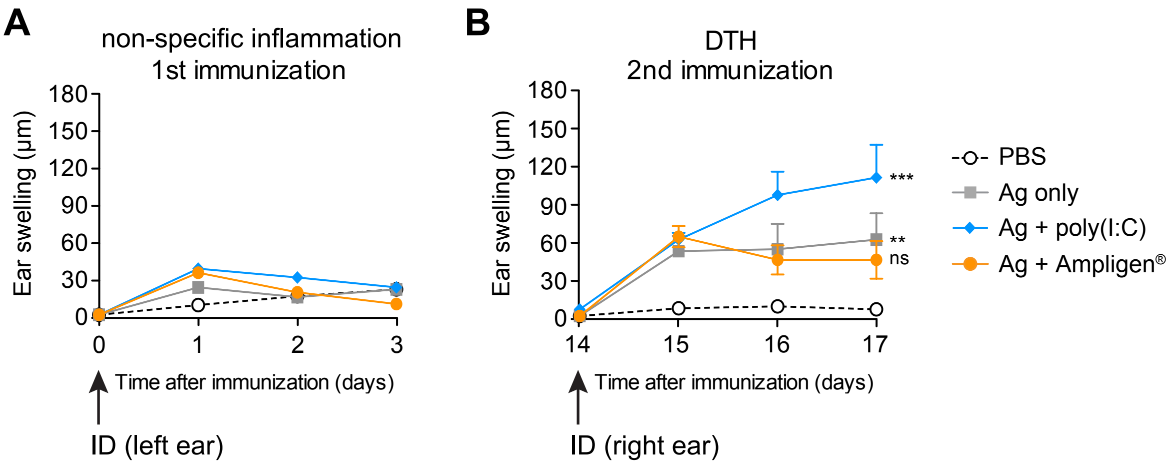

Supplemental Figure 2.

ID vaccination with HSV-2 gD + poly(I:C) does not result in non specific inflammation and promotes a DTH response. (A) Non specific inflammation was measured by ear swelling at various time points after the first ID injection with gD alone or together with Poly(I:C) or Ampligen®. (B) Skin DTH response was determined by ear swelling at various time points after the second ID injection performed on day 14 in mice previously immunized on day 0. Results are expressed as mean + SEM of ear swelling (µm) in 6 mice/group and are representative of one out of 2 independent experiments. Statistics using the two-way ANOVA test with Bonferroni multiple comparisons. Asterisks next to the group symbol depicts comparison with the PBS control group.
